# Machine learning approaches to predict hip fracture incidence: insights from the CHARLS dataset

**DOI:** 10.3389/fpubh.2025.1624843

**Published:** 2026-01-13

**Authors:** Yuexin Li, Yihua Shi

**Affiliations:** Department of Orthopaedics, Xiangyang No.1 People’s Hospital, Hubei University of Medicine, Xiangyang, China

**Keywords:** CHARLS, hip fracture, machine learning, predictive model, SHAP analysis

## Abstract

**Background:**

Hip fractures are a major health concern in the older adults, severely impacting patients’ quality of life and straining healthcare systems. With China’s aging population, their incidence is projected to increase. Thus, developing effective prediction models to identify high-risk individuals is essential for prevention.

**Objective:**

The aim of this study was to develop and validate a reliable and accurate machine learning-based predictive model for hip fracture incidence to improve the prediction of the risk of hip fracture in community residents.

**Methods:**

Data were obtained from the China Health and Retirement Longitudinal Study (CHARLS), encompassing 21,095 individuals aged 45 years and older, of whom 616 reported hip fractures. Baseline data from these participants were utilized to examine 34 metrics, including demographic characteristics, lifestyle, health status, and mental health and cognitive functioning scores. Ten machine learning algorithms, including Random Forest (RF), Adaptive Boosting (AdaBoost), and Decision Tree (DT) machine learning techniques were used to analyze and determine the optimal model. The performance of the predictive models was evaluated by area under the receiver operating characteristic curve (AUC), sensitivity, specificity and F1 score. The SHapley Additive exPlanation (SHAP) interpretation was utilized in identifying the key influencing factors and individual heterogeneity was explained through instance level analysis.

**Results:**

After addressing class imbalance using a class-weighting technique, we found that the Random Forest model performed the best, with an AUC of 0.93, with high sensitivity, specificity, and F1 score. Metabolic Equivalent of Task (MET), age, fall down, drinking, cognitive functioning scores, sleep duration of nap after lunch, residence, total sleep duration, and marital status were the key predictors. The model demonstrated favorable predictive performance in both the internal and external validation cohorts, indicating that the model is optimal.

**Conclusion:**

The machine learning-based predictive model developed in this study demonstrated strong predictive performance for incident hip fractures over a 7-year period. By incorporating readily available, modifiable lifestyle factors, the model serves as a promising tool for identifying individuals at high risk. It provides a scientific basis for developing early intervention strategies, but requires further prospective validation before clinical implementation.

## Graphical abstract

**Figure:**
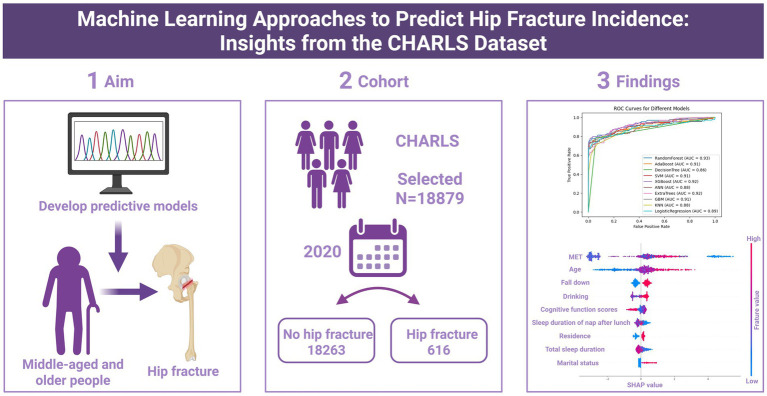

## 1 Background

Hip fracture is a common health problem among the older adults and its incidence is on the rise as the population ages (1). Hip fractures not only severely affect the quality of life of patients, but also place a heavy burden on the healthcare system (2). Falls are the most common cause of hip fractures, especially among older adults with osteoporosis. In addition, as we age, strength and balance decline and bones become more fragile, making older adults more susceptible to falls and fractures (3). Treatment of hip fractures usually requires surgery, but surgery does not guarantee a full recovery, and many patients require long-term care and rehabilitation after surgery (4). This imposes a considerable burden on individuals, families and society as a whole. Therefore, there is an urgent demand for a method that can effectively recognize people at high risk of developing hip fractures and that can provide an effective intervention strategy to reduce the incidence of hip fracture.

CHARLS is a survey program that aims to collect high-quality microdata on households and individuals of middle-aged and older people aged 45 and above in China (5). It provides a scientific basis for analyzing China’s aging population, promoting interdisciplinary research, and providing a basis for policy development, as well as being a key resource for the study of hip fracture (6). The application of machine learning techniques has enabled researchers to identify and predict risk factors for disease onset from CHARLS data, which is critical for early intervention and preventive measures (7). Machine learning models are able to process and analyze large amounts of complex data, identifying key factors that influence hip fracture risk to support clinical decision-making.

Currently, predictive risk factors for hip fracture include age, advanced age, western region, lower education, underweight, smoking, self-reported chronic lung disease, heart disease, diabetic course of stroke, history of arthritis, mental status, and glucocorticoids, among others (8–11). Although a number of tools and methods exist for assessing hip fracture risk, there is a lack of validated tools for early identification of those individuals most likely to benefit from preventive measures. For example, the FRAX tool is a tool that predicts the probability of hip fracture in cancer patients over the next 10 years based on clinical risk factors and bone density, but it needs to be optimized and personalized even further for different populations (12). The Garvan Fracture Risk Calculator (FRC) is a nomogram tool that can be used to calculate the 5- and 10-year absolute risk of hip fracture, which was developed from data from the Dubbo Osteoporosis Epidemiology Study cohort in Australia (13). Both of these previously developed tools are currently the more authoritative tools for fracture risk prediction. However, they focus primarily on factors related to age, clinical indicators, and Bone Mineral Density, and do not explore the potential of indicators related to sociodemographic characteristics, lifestyle, health status, and mental health. In addition, since both tools were developed for populations adapted to Western populations, they may not be adapted to non-Western populations. Thus machine learning enables the development of community cohort hip fracture prediction models adapted to non-Western populations in these more readily available metrics, able to be independent of imaging and laboratory markers that are only available after hospitalization. This provides an important direction for the development of hip fracture risk tools and also resolves the differences in predictive modeling between non-Western and Western populations.

This study developed and validated a machine learning-based hip fracture risk prediction model. Ten machine learning algorithms were used to analyze and identify the Random Forest model as the best model with high sensitivity, specificity, and F1 score. Key predictors were identified, including MET, age, fall down, drinking, cognitive functioning scores, sleep duration of nap after lunch, residence, total sleep duration, and marital status. These findings contribute to the development of more accurate predictive tools and provide guidance for clinical practice to better identify those individuals at high risk and take preventive measures. The study’s predictive modeling could help reduce the incidence of hip fractures, lower associated healthcare costs, and improve the quality of life for older adults. Through early identification and intervention, long-term disability and reduced quality of life due to hip fracture in older adults can be reduced, while also reducing the burden on families and society. In addition, the study emphasizes the importance of improving lifestyle and environmental factors in the prevention of hip fracture, providing a scientific basis for the development of public health policies in China.

## 2 Methods

### 2.1 Study population

CHARLS is a national research program implemented by the National Development Research Institute of Peking University since 2011, focusing on the health, retirement, and socioeconomic status of the middle-aged and older adult population. CHARLS employs computer-assisted personal interview (CAPI), physical measurements, and blood sample collection, and ensures that the samples are nationally representative through multi-stage probability proportional to size (PPS) random sampling. The questionnaire covered socio-demographic, psychological status, and health status, including information on age, gender, education, marriage, physical activity, chronic disease, mental health, cognitive functioning, economic status, pension, and health care coverage. The study population is middle-aged and older adults aged 60 years and older, covering several provinces across the country, including both urban and rural areas, to ensure the breadth and diversity of the data. The data for this study were derived from the 2020 wave of the CHARLS database and approved by the Biomedical Ethics Committee of Peking University (IRB00001052-11015.1.2). All participants signed an informed consent form, and those who could not read and write gave their consent by verbal explanation followed by a handprint. The CHARLS project website^1^ provides information on study methods, data access, and publications (14). We used the 2011 wave as our baseline and the 2018 wave for follow-up outcome ascertainment. Inclusion criteria for this study were: (1) Age 45 years and above; (2) Informed consent. Exclusion criteria: (1) age below 45 years; (2) missing values with >10% in the interested variables.

### 2.2 External validation

To assess the model’s generalizability, we conducted external validation using an independent cohort. This cohort was prospectively enrolled at Xiangyang No.1 People’s Hospital from 2025. Inclusion and exclusion criteria were designed to mirror those of the CHARLS cohort (e.g., age ≥ 45, no prior hip fracture at enrollment). All variables were collected by trained clinical research coordinators using standardized case report forms, ensuring measurement consistency. The external cohort consisted of 1,200 participants, among whom 37 incident hip fracture events were recorded, corresponding to an event rate of 3.1%, which is comparable to the CHARLS training cohort. The research protocol was approved by the Medical Ethics Committee of the Xiangyang No.1 People’s Hospital.

### 2.3 Hip fracture

The primary outcome of this study was incident hip fracture occurring between the 2011 baseline and the 2018 follow-up. At the 2018 follow-up wave, participants were asked, “Have you had a hip fracture since your last interview (in 2011)?” after being given a detailed description of the hip bone’s location. Responses were recorded as “yes” or “no” in a self-report format. This approach ensures that the outcome occurred after the measurement of baseline predictors. This method of interviewing was designed to increase the accuracy of the respondents’ answers and to ensure the reliability of the data.

### 2.4 Covariates

Data on a comprehensive set of indicators were extracted from the 2020 CHARLS database on indicators including sociodemographic data, health behavioral factors, chronic diseases (hypertension, diabetes mellitus, dyslipidemia, heart disease, stroke, cancer, lung disease, arthritis, renal disease, liver disease, digestive system diseases, Parkinson’s disease, asthma, psychiatric disorders, and memory disorders), physical activity (MET and intensity of activity), falls, cognitive function scores, depression scale, ability to perform activities of daily living (ADL), and instrumental activities of daily living scale (IADL) indicator data, were used as covariates. These data were collected to ensure that the study was able to comprehensively analyze the factors affecting hip fracture.

- For the sociodemographic data, we selected variables including age, gender (female or male), Residence (urban or rural), educational attainment (illiterate or primary, middle school, or college and above), and marital status (married, separated, divorced, widowed, or unmarried).
- For health behavioral factors and chronic diseases, we selected variables including smoking status (never, current and quit), drinking (never, current and quit), sleep duration (sleep duration at night, sleep duration of nap after lunch and total sleep duration) and chronic diseases (yes or no).
- For physical activity and other covariates, we selected variables including intensity of activity (light activity, moderate activity, and heavy activity), falls (yes or no), cognitive function scores, depression scale, ADL and IADL. In terms of activity intensity, light activities were characterized by minimal physical exertion, such as walking. Moderate activities involved a moderate level of physical exertion, including participation in club-organized events or practicing Tai Chi. Vigorous activities were defined by a high degree of physical exertion, encompassing tasks such as gardening, constructing walls, and engaging in workouts. Data on the duration of daily activities and the specific days of the week on which these activities occurred were collected from the subjects. MET = (8.0 × Weekly Frequency (days) × Time per Day (minutes)) + (4.0 × Weekly Frequency (days) × Time per Day (minutes)) + (3.3 × Weekly Frequency (days) × Time per Day (minutes)) (15).

### 2.5 Model development and feature screening

The dataset comprised 18,263 subjects without hip fractures and 616 subjects with hip fractures. To mitigate the risk of overfitting and enhance the model’s generalization capabilities and predictive performance, we randomly partitioned the dataset into a training set (80%) and a validation set (20%). Missing data were addressed through median interpolation. To address the significant class imbalance (~3.3% fracture incidence), a class-weighting technique (specifically, class_weight = ‘balanced’) was employed across all machine learning algorithms. This method assigns higher weights to the minority class (hip fracture), forcing the models to pay more attention to correctly identifying these critical cases. Initially, ten machine learning models (AdaBoost, DT, RF, support vector machine (SVM), eXtreme Gradient boosting (XGboost), Artificial Neural Network (ANN), Extra Tree (ET), Gradient Boosting Machine (GBM), K-Nearest Neighbor (KNN) and Logistic Regression (LR)) were employed to train the 34 features within the training set. Hyperparameter tuning and model stability assessment were conducted using a 10-fold cross-validation strategy exclusively on the training set. The optimal model was subsequently identified by evaluating and comparing AUC, sensitivity, specificity, positive predictive value (PPV), negative predictive value (NPV), accuracy, and F1 score for each model. The 34 features were systematically reduced utilizing the optimal model, and critical features were identified based on variations in AUC, sensitivity, specificity, and F1 score. To guarantee that the optimal model demonstrates robust performance on previously unseen data, we conducted a 10-fold cross-validation on the dataset. To assess clinical benefit, we employed decision curve analysis (DCA) to compute the net benefit across various probability thresholds. To enhance the interpretability of machine learning outcomes, we present the SHAP method, which is grounded in the Shapley value concept from game theory. This approach assigns an importance value to each feature within the model, conceptualizing each feature as a participant in a cooperative game. By calculating the contribution of each feature to the final prediction, SHAP quantifies the importance of each feature, thereby offering a consistent and equitable interpretation of the model’s predictions (16). The application of SHAP values facilitated the reduction of the feature set from 34 to 9 features, thereby enhancing the model’s predictive performance. SHAP offers both global and local interpretative frameworks. The global interpretation yields consistent attribution values for each feature, demonstrating their association with hip fracture. In contrast, local explanations provide predictions tailored to individual cases.

### 2.6 Statistical analysis

Data analysis was performed using Python version 3.6.5 and SPSS statistical software version 23.0. Biased continuous variables were described by median and interquartile range and were compared through the Mann–Whitney U test or the Kruskal-Wallis H test. Categorical variables were presented as counts and percentages, with comparisons made using the chi-square test or Fisher’s exact test. Covariate adjustment was carried out using ANCOVA. *p* values ≤0.05 were deemed to indicate statistical significance.

## 3 Results

### 3.1 Study population and design

**Figure 1:**
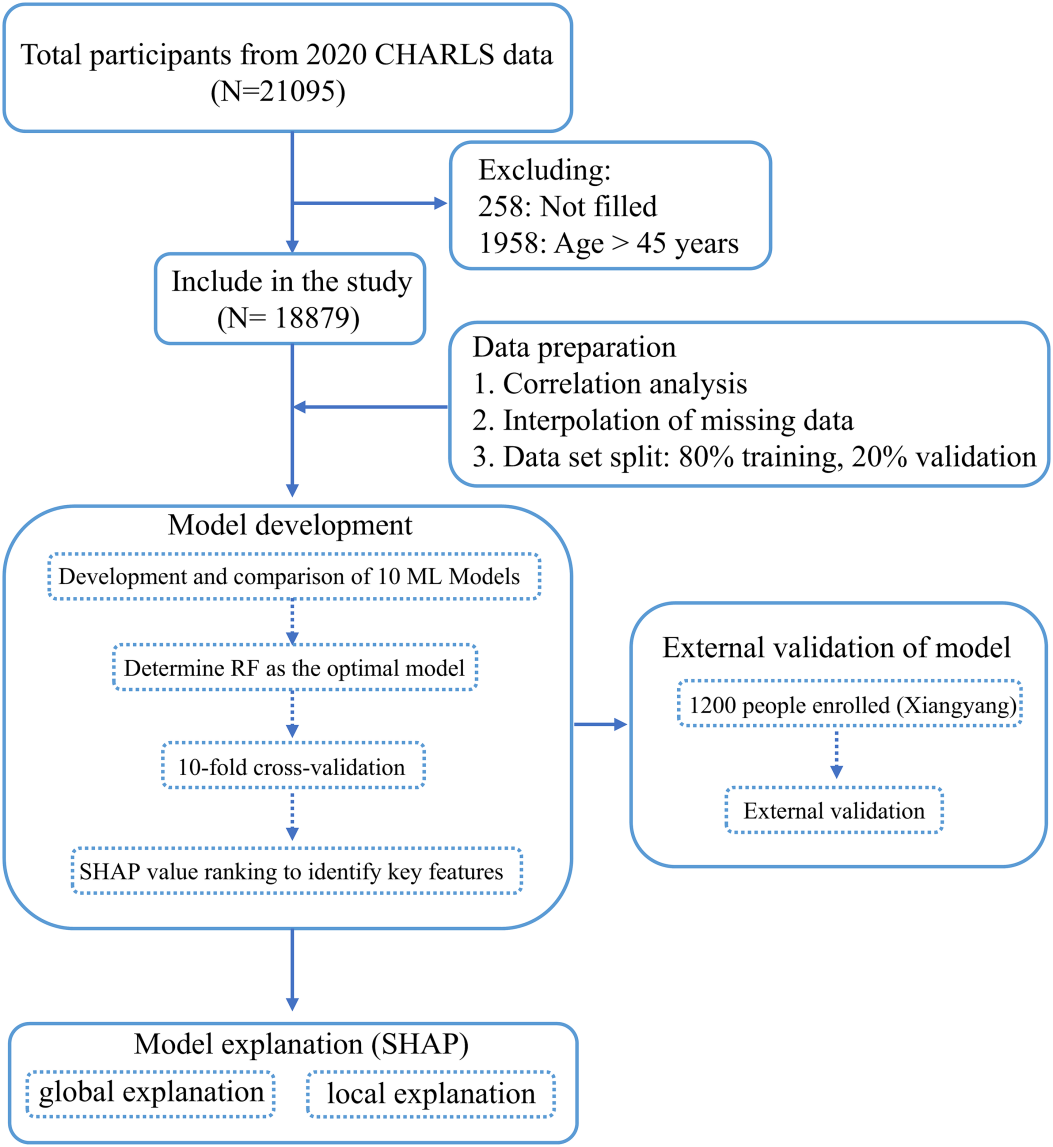
Flow chart of the study design.

In this study, we conducted a screening of 21,095 participants from the CHARLS database to develop a predictive model for hip fracture risk. Initially, we excluded 258 participants due to incomplete data, followed by the exclusion of 1,958 individuals under the age of 45. Consequently, 18,879 participants met the eligibility criteria and were included in the analysis. Among these, 616 participants self-reported a history of hip fracture. To optimize our prediction model, we split eligible participants into training and validation sets and gathered an independent cohort of 1,200 adults to test the model’s generalizability. The study design is outlined in Figure 1.

### 3.2 Analysis of factors linked to hip fractures in middle-aged and older adults

Table 1 shows that compared to the non-hip fracture group, the hip fracture group was older, lived in rural areas, low education level, lived alone, had shorter night and total sleep times, more underlying diseases, lower MET and activity levels, were more prone to falls, had lower cognitive function, higher depression scores, and reduced ability in daily activities (*p* < 0.001). There were no significant differences in sex ratio, nap time, cancer, or strenuous exercise between the groups (*p* > 0.05).

**Table 1:** The baseline characteristics of the study population between non-hip fracture and hip fracture.

Variables	Hip fracture		Statistic	*P*-value
	No (*n* = 18,263)	Yes (*n* = 616)		
Age	61 (54,68)	66 (57,74)	−9.796	<0.001
Male	8,647 (47.35%)	271 (43.99%)	2.689	0.101
Residence			29.295	<0.001
Urban	6,659 (36.46%)	159 (25.81%)		
Rural	11,604 (63.54%)	457 (74.19%)		
Educational			49.858	<0.001
Literate or primary school	11,815 (64.69%)	483 (78.41%)		
Middle school	6,035 (33.05%)	127 (20.62%)		
College and above	413 (2.26%)	6 (0.97%)		
Marital status			68.508	<0.001
Married	15,355 (84.08%)	448 (72.73%)		
Separated	77 (0.42%)	4 (0.65%)		
Divorced	237 (1.30%)	4 (0.65%)		
Widowed	2,489 (13.63%)	154 (25%)		
Unmarried	105 (0.58%)	6 (0.97%)		
Smoking			3.983	0.136
No	4,657 (25.5%)	138 (22.40%)		
Current	2,441 (13.37%)	94 (15.26%)		
Quit	11,165 (61.13%)	384 (62.34%)		
Drinking			4.317	0.115
No	10,514 (57.57%)	380 (61.69%)		
Current	6,550 (35.87%)	197 (31.98%)		
Quit	1,199 (6.57%)	39 (6.33%)		
Sleep duration at night	6 (5,7)	6 (4,7)	4.441	<0.001
Sleep duration of nap after lunch	30 (0,60)	30 (0,60)	−0.429	0.668
Total sleep duration	420 (330,480)	390 (300,480)	3.438	0.001
Parkinson’s disease	239 (1.31%)	26 (4.22%)	36.515	<0.001
Hypertension	7,308 (40.02%)	304 (49.35%)	21.582	<0.001
Dyslipidaemia	4,819 (26.39%)	198 (32.14%)	10.119	0.001
Hyperglycaemia	2,681 (14.68%)	130 (21.10%)	19.405	<0.001
Cancer	457 (2.50%)	22 (3.57%)	2.754	0.097
Chronic lung diseases	2,574 (14.09%)	141 (22.89%)	37.440	<0.001
Hepatic disease	1,299 (7.11%)	75 (12.175%)	22.632	<0.001
Heart disease	3,775 (20.67%)	178 (28.896%)	24.357	<0.001
Stroke	1,277 (6.99%)	92 (14.94%)	55.897	<0.001
Kidney disease	1874 (10.26%)	95 (15.42%)	16.990	<0.001
Digestive system disease	5,723 (31.34%)	258 (41.88%)	30.624	<0.001
Emotional and mental disorders	529 (2.90%)	41 (6.66%)	28.761	<0.001
Memory related disease	750 (4.11%)	46 (7.47%)	16.667	<0.001
Arthritis	6,950 (38.06%)	353 (57.31%)	93.098	<0.001
Asthma	1,081 (5.92%)	79 (12.83%)	49.276	<0.001
MET	4,932 (2,628,9,492)	4,155 (1138.5,9,972)	3.454	0.001
Vigorous exercise	6,498 (35.58%)	212 (34.42%)	0.353	0.553
Moderate exercise	10,204 (55.87%)	274 (44.48%)	31.313	<0.001
Mild exercise	14,098 (77.19%)	419 (68.02%)	28.234	<0.001
Fall down	6,024 (32.99%)	421 (68.34%)	331.367	<0.001
Cognitive function scores	12 (7,15)	9 (4,13)	9.525	<0.001
Depression scale	7 (4,12)	9 (6,16)	−9.165	<0.001
ADL	0 (0,0)	0 (0,2)	−15.208	<0.001
IADL	0 (0,0)	0 (0,4)	−13.301	<0.001

### 3.3 Model development and performance comparison

**Figure 2:**
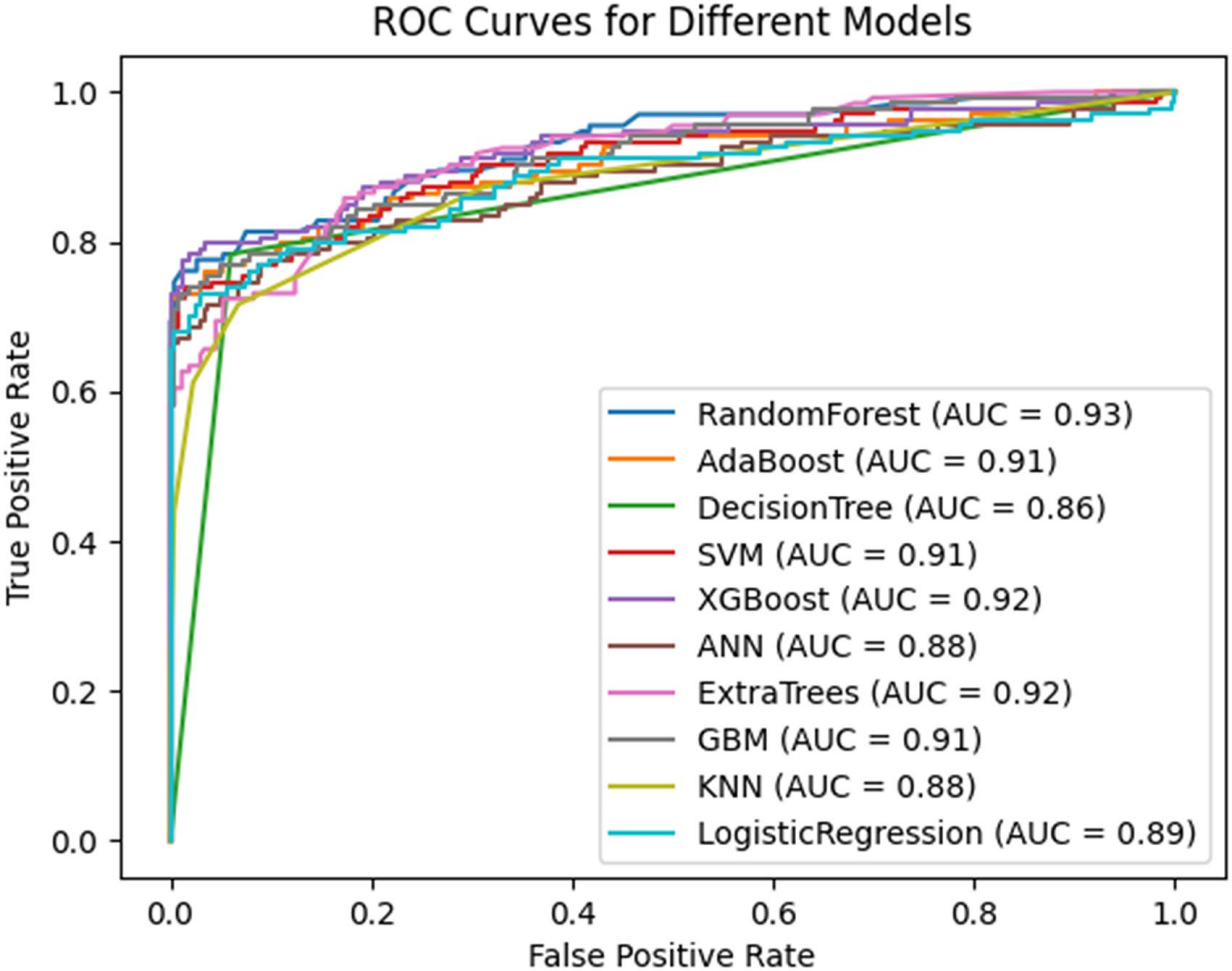
Performance of 10 machine learning models for predicting hip fractures. ROC curve (the *x*-axis indicates the false positive rate, and the *y*-axis represents the true positive rate).

To minimize overfitting and enhance the model’s generalization and prediction performance, we randomly split the dataset into a training set (15103) and a validation set (3776), and compared these with clinical baseline data from 1,200 volunteers at the Xiangyang No.1 People’s Hospital (Table 2). Table 2 indicates that most variables showed no significant differences among the three groups (*p* > 0.05). We utilized CHARLS data to create 10 machine learning models, including Random Forest, AdaBoost, Decision Tree, SVM, XGboost, ANN, Extra Trees, GBM, KNN and Logistic Regression, to predict hip fracture incidence in individuals over 45. As detailed in Table 3, multiple models achieved high AUCs. However, the Random Forest (RF) model was selected as the optimal model due to its superior overall robustness across multiple key metrics. Specifically, it achieved the highest or near-highest values for AUC (0.93), Accuracy (0.97), and, crucially, the F1-Score (0.55). The F1-Score, which balances precision and recall, is a particularly important indicator of performance in imbalanced datasets, making the RF model the most effective and reliable choice. To illustrate, we plotted the ROC curves for all 10 models (Figure 2).

**Table 2:** Comparison of baseline characteristics among the training, internal validation, and external validation cohorts.

Variables	Training (*n*=15103)	Internal validation (*n*=3776)	External validation (*n*=1200)	Statistic	*P*-value
Age	61 (54,68)	62 (54,69)	61 (54,69)	0.443	0.801
Male	7,104 (47.04%)	1814 (48.04%)	569 (47.42%)	1.234	0.539
Residence				1.629	0.443
Urban	5,481 (36.29%)	1,337 (35.41%)	420 (35%)		
Rural	9,622 (63.71%)	2,439 (64.59%)	780 (65%)		
Educational				5.882	0.208
Literate or Primary School	9,796 (64.86%)	2,502 (66.26%)	757 (63.08%)		
Middle school	4,966 (32.88%)	1,196 (31.67%)	420 (35%)		
College and above	341 (2.26%)	78 (2.07%)	23 (1.92%)		
Marital status				3.380	0.908
Married	12,644 (83.72%)	3,159 (83.66%)	1,004 (83.67%)		
Separated	70 (0.46%)	11 (0.29%)	4 (0.33%)		
Divorced	189 (1.25%)	52 (1.38%)	16 (1.33%)		
Widowed	2,114 (14.00%)	529 (14.01%)	170 (14.17%)		
Unmarried	86 (0.57%)	25 (0.66%)	6 (0.5%)		
Smoking				2.460	0.652
No	3,835 (25.39%)	960 (25.42%)	282 (23.5%)		
Current	2025 (13.41%)	510 (13.51%)	159 (13.25%)		
Quit	9,243 (61.2%)	2,306 (61.07%)	759 (63.25%)		
Drinking				3.185	0.527
No	8,687 (57.52%)	2,207 (58.45%)	694 (57.83%)		
Current	5,415 (35.85%)	1,332 (35.28%)	439 (36.58%)		
Quit	1,001 (6.63%)	237 (6.28%)	67 (5.58%)		
Sleep duration at night	6 (5,7)	6 (5,7)	6 (5,7)	6.409	0.041
Sleep duration of nap after lunch	30 (0,60)	30 (0,60)	30 (0,60)	4.746	0.093
Total sleep duration	420 (330,480)	420 (330,480)	420 (330,480)	6.217	0.045
Parkinson’s disease	216 (1.43%)	49 (1.30%)	14 (1.17%)	0.850	0.654
Hypertension	6,086 (40.30%)	1,526 (40.41%)	491 (40.92%)	0.184	0.912
Dyslipidaemia	4,010 (26.55%)	1,007 (26.67%)	336 (28%)	1.194	0.550
Hyperglycaemia	2,238 (14.82%)	573 (15.18%)	180 (15%)	0.314	0.855
Cancer	376 (2.49%)	103 (2.73%)	22 (1.83%)	3.002	0.223
Chronic lung diseases	2,185 (14.47%)	530 (14.04%)	181 (15.08%)	0.906	0.636
Hepatic disease	1,091 (7.22%)	283 (7.50%)	97 (8.08%)	1.405	0.495
Heart disease	3,169 (20.98%)	784 (20.76%)	252 (21%)	0.091	0.956
Stroke	1,117 (7.40%)	252 (6.67%)	95 (7.92%)	3.069	0.216
Kidney disease	1,595 (10.56%)	374 (9.91%)	120 (10%)	1.619	0.445
Digestive system disease	4,796 (31.76%)	1,185 (31.38%)	384 (32.03%)	0.256	0.880
Emotional and mental disorders	442 (2.93%)	128 (3.39%)	27 (2.25%)	4.561	0.102
Memory related disease	650 (4.30%)	146 (3.87%)	48 (4%)	1.565	0.457
Arthritis	5,856 (38.77%)	1,447 (38.32%)	468 (39%)	0.309	0.857
Asthma	915 (6.06%)	245 (6.489%)	76 (6.33%)	1.036	0.596
MET	4,860 (2,628,9,576)	4,944 (2,376,9,438)	4,854 (2043,8503.5)	2.951	0.229
Vigorous exercise	5,358 (35.48%)	1,352 (35.81%)	432 (36%)	0.246	0.884
Moderate exercise	8,380 (55.49%)	2098 (55.56%)	672 (56%)	0.121	0.941
Mild exercise	11,641 (77.078%)	2,876 (76.17%)	928 (77.33%)	1.538	0.464
Fall down	5,154 (34.13%)	1,291 (34.19%)	420 (35%)	0.378	0.828
Cognitive function scores	12 (7,15)	12 (6,15)	12 (7,16)	3.189	0.203
Depression scale	7 (4,12)	7 (4,12)	7 (4,11)	3.729	0.155
ADL	0 (0,0)	0 (0,0)	0 (0,0)	2.905	0.234
IADL	0 (0,0)	0 (0,1)	0 (0,1)	1.444	0.486

**Table 3:** Performance of the ML models for hip fracture prediction.

Model	AUC	Accuracy	Precision	Recall	F1 score	Specificity	PPV	NPV
Random Forest	0.93	0.91	0.99	0.73	0.84	1.00	0.99	0.88
AdaBoost	0.91	0.89	0.92	0.73	0.82	0.97	0.92	0.88
Decision Tree	0.86	0.89	0.87	0.78	0.82	0.94	0.87	0.90
SVM	0.91	0.89	0.98	0.69	0.81	0.99	0.98	0.87
XG boost	0.92	0.91	1.00	0.73	0.84	1.00	1.00	0.88
ANN	0.88	0.87	0.87	0.72	0.79	0.95	0.87	0.87
Extra Trees	0.92	0.84	1.00	0.52	0.69	1.00	1.00	0.81
GBM	0.91	0.90	0.93	0.75	0.83	0.97	0.93	0.88
KNN	0.88	0.85	0.93	0.61	0.74	0.98	0.93	0.83
Logistic Regression	0.89	0.88	0.93	0.70	0.80	0.97	0.93	0.87

### 3.4 Simplification of model features

**Figure 3:**
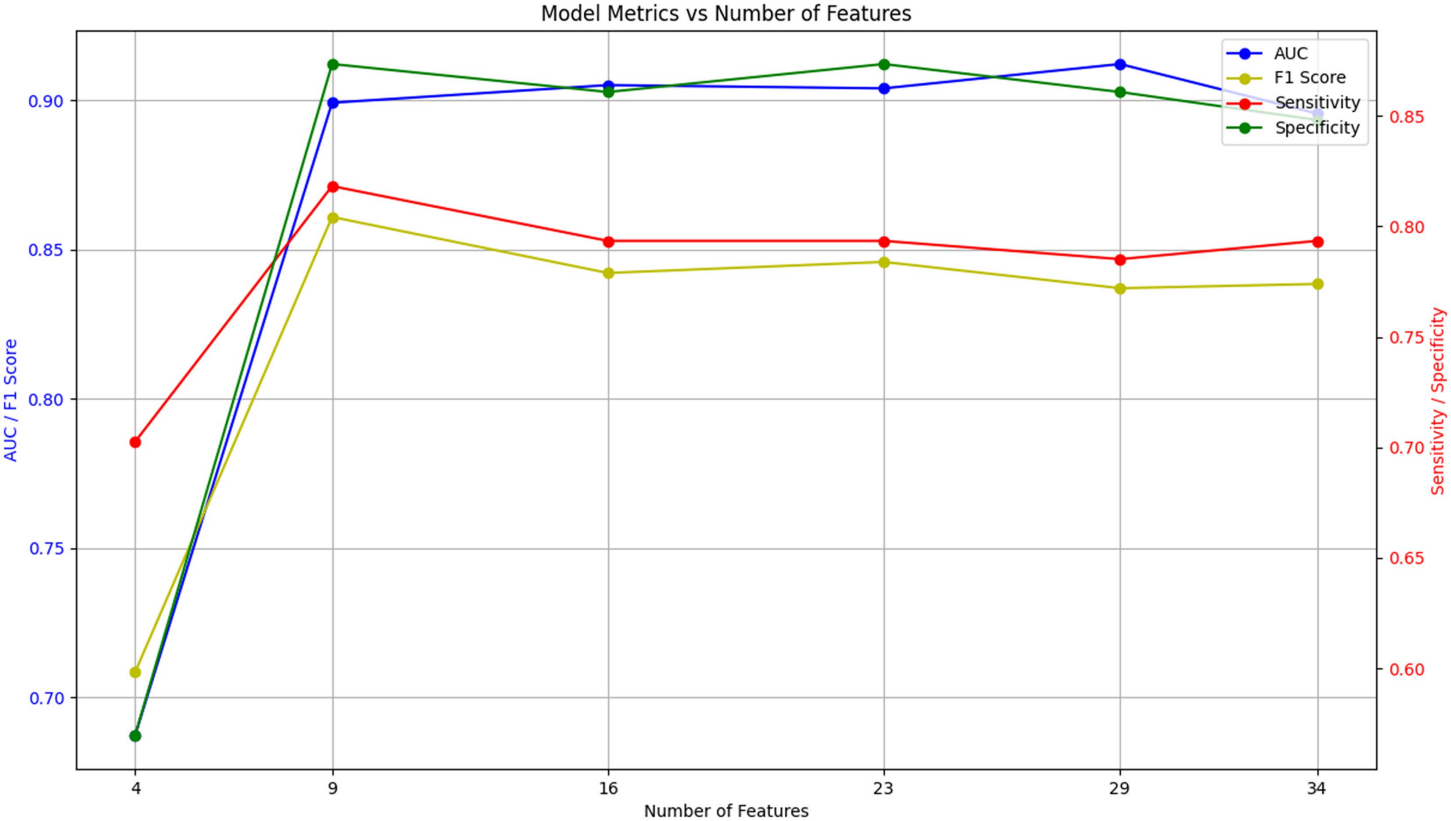
Simplification of model features. AUC for different quantitative characterization models.

To better generalize this predictive model efficiently, we need to simplify our initial input of 34 covariates to find the key few feature variables, and we plotted the AUC of the RF model for different numbers of features. Figure 3 indicates that the AUC value stays stable as features reduce from 34 to 9, but drops sharply below 9 features, suggesting 9 features are needed for strong predictive performance. Table 4 confirms that the model with 9 features significantly outperforms the one with 4 features.

**Table 4:** Performance metrics of RF models with different quantitative characteristics.

Features	AUC	Sensitivity	Specificity	F1 score
4	0.7624	0.7695	0.5405	0.7856
9	0.9602	0.9331	0.8739	0.9401
16	0.9602	0.9257	0.9279	0.9468
23	0.9667	0.9257	0.9550	0.9522
29	0.9786	0.9257	0.9640	0.9540
34	0.9766	0.9257	0.9640	0.9540

### 3.5 10-fold cross-validation and external validation

**Figure 4:**
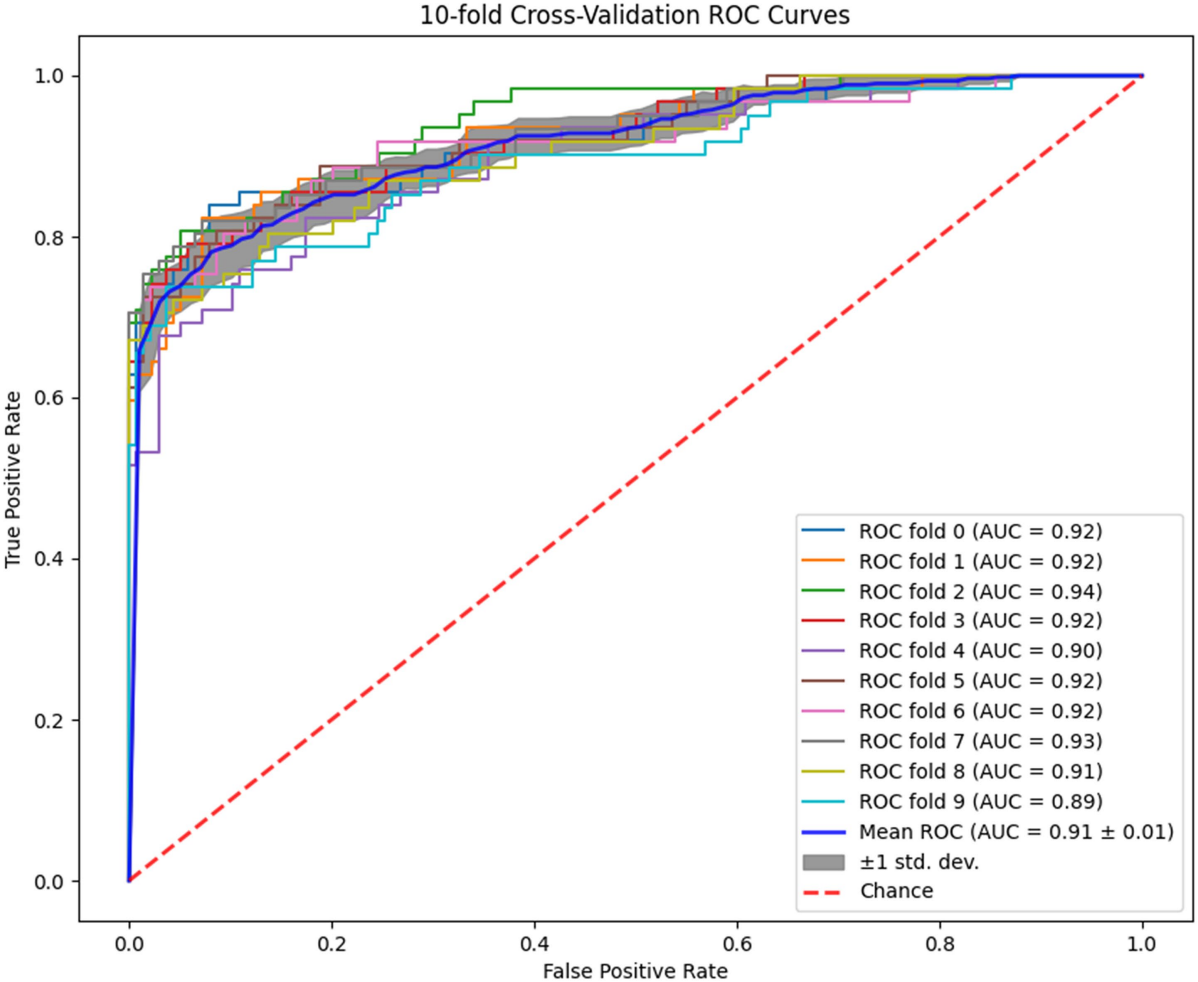
10-fold cross-validation of RF model.

**Figure 5:**
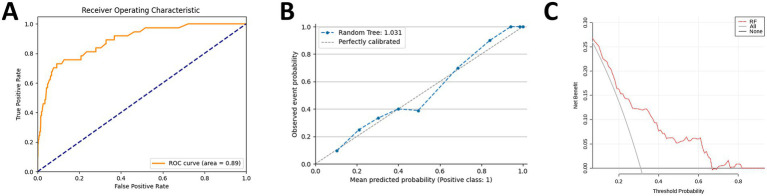
About external validation sets: **(A)** ROC curve; **(B)** calibration; **(C)** decision curve analysis.

To confirm the RF model’s strong performance on unseen data, we conducted 10-fold cross-validations, yielding an average AUC of 0.91 ± 0.01 (Figure 4). In external validation, the AUC was 0.89 (Figure 5A). To provide a more stringent evaluation in the context of class imbalance, we also generated a Precision-Recall (PR) curve. The PR curve demonstrates that the model maintains high precision across a range of recall values, with an area under the PR curve (AUPRC) is 0.867, confirming its effectiveness for the rare positive class (Supplementary Figure S1). Both internal and external results indicate the RF model performs well. An external validation set was employed to develop the clinical calibration curves, revealing that the predicted probabilities of hip fracture risk were in strong concordance with the observed outcomes (Figure 5B). To assess model calibration, we generated a calibration plot using the external validation set, which demonstrated excellent agreement between the model’s predicted probabilities and the observed frequencies of hip fracture (Supplementary Figure S2). Further supporting this, the Hosmer-Lemeshow test yielded a non-significant *p*-value (*p* = 0.48), providing no evidence of poor calibration. Furthermore, the Brier score was utilized to evaluate the model’s overall calibration, yielding a score of 0.0103, which signifies excellent calibration performance. Through the application of Decision Curve Analysis (DCA) on an external validation set (Figure 5C), we demonstrate the net benefit of the Random Forest (RF) model within a specified threshold probability interval (0.01 to 0.81). Within this range, the model significantly enhances clinical outcomes compared to the strategies of intervening in all patients or not intervening at all. This characteristic of the RF model indicates its substantial utility in clinical settings, where it can aid clinicians in making more accurate treatment decisions based on predicted hip fracture risk.

### 3.6 SHAP analysis

**Figure 6:**
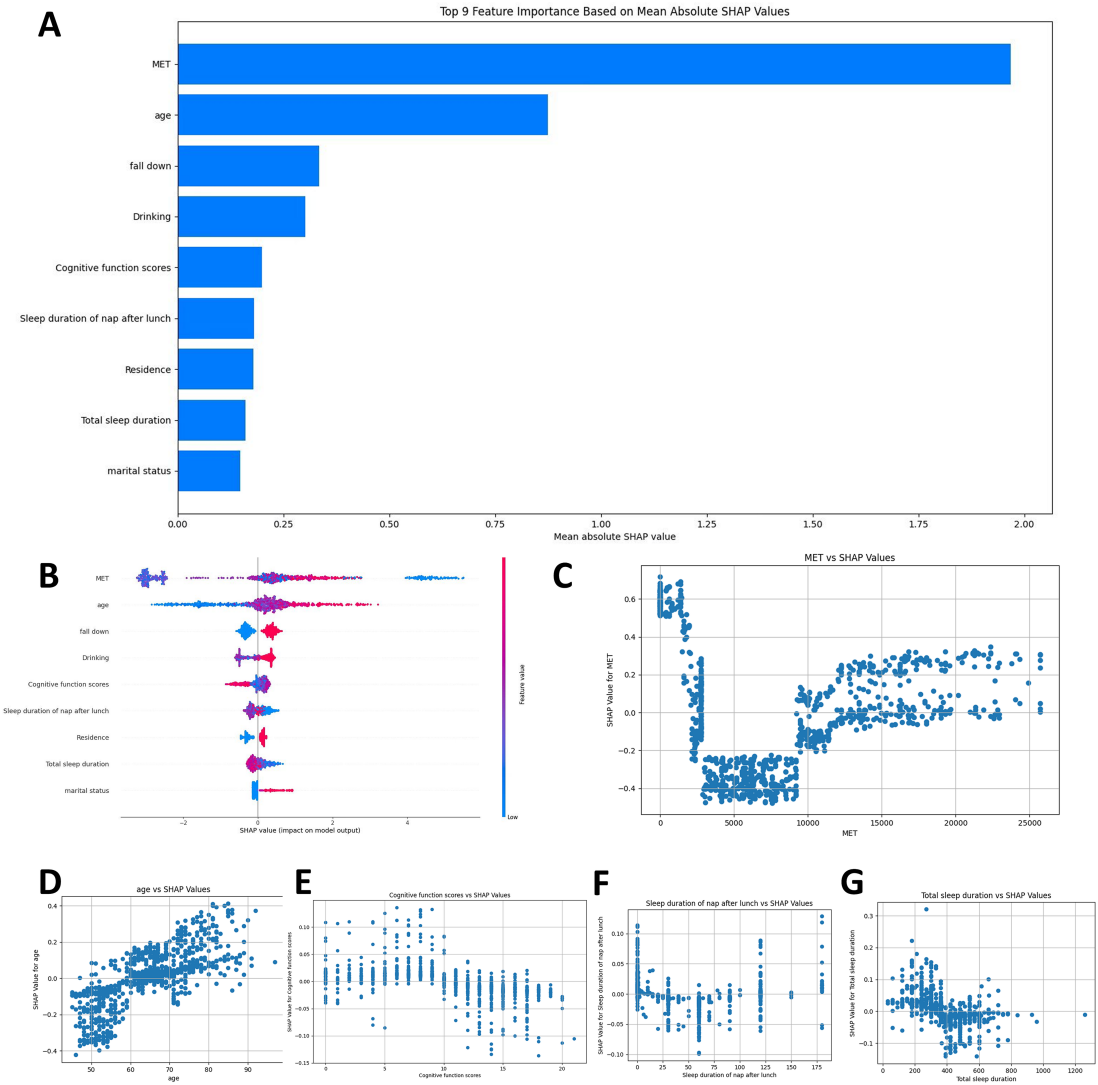
Global model explanation using SHAP analysis. **(A)** Features are ranked by their mean absolute SHAP value, indicating overall model impact. **(B)** The SHAP beeswarm plot shows the impact of each feature on the model output for every individual. Each point is a person; its position on the *x*-axis shows its impact on risk. The color indicates the feature’s value (red = high, blue = low). **(C–G)** SHAP dependence plots illustrate the marginal effect of the five most important predictors. These plots reveal the relationship between a feature’s value and its contribution to the predicted risk, highlighting non-linear trends and thresholds.

**Figure 7:**
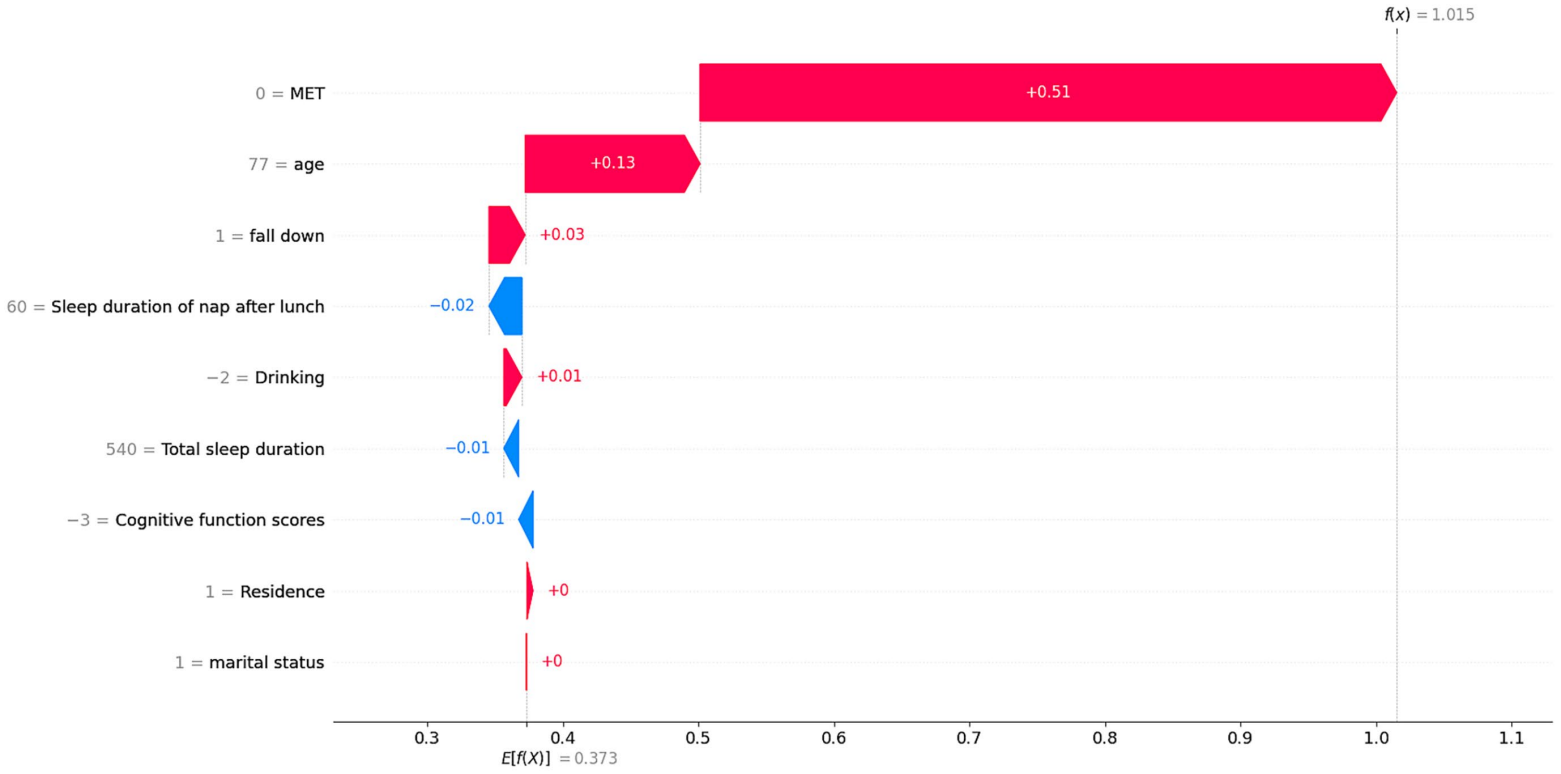
The local interpretation of a patient with a hip fracture.

To elucidate the contribution of the selected variables in predicting hip fracture risk factors, we employed the SHAP analysis to quantify their respective influences. In our visual representation, we ranked the features according to their SHAP values across all samples and highlighted the 9 most influential features (MET, age, fall down, drinking, cognitive function scores, sleep duration of nap after lunch, residence, total sleep duration and marital status). The vertical axis of the graph denotes the importance ranking of these features, while the horizontal axis illustrates the SHAP value associated with each feature (Figure 6A). To provide a more comprehensive visual representation of the distribution of each sample across the features, we generated a Beeswarm Plot as part of the SHAP analysis (Figure 6B). Each point on the graph corresponds to an individual sample, and the points are organized into vertical stacks to facilitate the observation of the overall sample distribution. Furthermore, we employed a color gradient to denote the magnitude of the feature values, with red representing high values and blue indicating low values. This color coding facilitates a more intuitive comprehension of the specific influence of each eigenvalue on the model’s predictions. In Figure 6B, the red color indicates higher risk values, while the blue color signifies lower risk values. The displacement of each point from the midline suggests an increased propensity for hip fractures. Notably, the AGE feature demonstrates a heightened risk of hip fractures associated with advancing age. Furthermore, the MET trait exhibits the most significant impact on hip fracture risk, revealing a distinct polarized trend. To enhance the visualization of the impact of individual traits on the prediction model’s output, we generated SHAP dependency plots for variables including MET, age, cognitive function scores, sleep duration of nap after lunch and total sleep duration (Figures 6C–G). Figure 6C illustrates that individuals with MET values ranging from 3,000 to 9,000 exhibit a reduced risk of hip fracture. In contrast, those with MET values below 3,000 and above 9,000 demonstrate a progressively increased risk of hip fracture, which is an interesting finding. The likelihood of experiencing a hip fracture escalates with advancing age (Figure 6D). Individuals exhibiting cognitive function scores exceeding 10 demonstrated a reduced risk of hip fracture (Figure 6E). There is a progressively elevated risk of hip fracture associated with sleep duration of nap after lunch exceeding 60 min (Figure 6F). Sleeping more than 400 min at night is effective in reducing the risk of hip fractures occurring (Figure 6G). The local interpretation analyzes how individual-specific input data can be combined to make predictions for a specific individual. The case of a patient with a hip fracture is illustrated in Figure 7. In summary, our findings indicate that excessive physical activity and inactivity, advanced age, incidence of falls, alcohol consumption, diminished cognitive function, prolonged lunch breaks, rural residency, inadequate total sleep duration, and solitary living are significant risk factors for hip fractures among middle-aged and older individuals.

## 4 Discussion

This research focuses on the development and validation of a machine learning-based predictive model designed to assess hip fracture risk among middle-aged and older adults within the Chinese population. By leveraging data from the CHARLS, the study employs various machine learning algorithms, with a particular emphasis on the Random Forest model. Additionally, SHAP analysis is utilized to identify significant risk factors. The outcome is a hip fracture prediction tool that is both accurate and interpretable, specifically tailored for applicability within the Chinese demographic. The ultimate objective is to mitigate the incidence of hip fractures through early identification and intervention of at-risk individuals. These findings offer significant insights into the predictors of hip fractures and illustrate the potential utility of machine learning in public health applications (17).

The development of our predictive model represents a substantial advancement in hip fracture risk assessment, with three key novel contributions. First, it is, to our knowledge, the first model for the Chinese population to integrate a wide array of non-invasive predictors, including lifestyle and mental health metrics, making it ideal for community screening. Second, through rigorous comparison and the use of SHAP analysis, we deliver a model that is both highly accurate and fully interpretable, revealing complex risk patterns like the U-shaped effect of MET. Third, it addresses the critical need for a validated, population-specific tool, as existing instruments were primarily developed on Western cohorts. Our RF model, achieving an AUC of 0.93, exhibits superior sensitivity, specificity, and F1 score in comparison to traditional models, thereby underscoring the potential of machine learning in predictive healthcare. The primary predictors identified include MET, age, fall down, drinking, cognitive function scores, sleep duration of nap after lunch, residence, total sleep duration and marital status. The significance of MET in our model highlights the dual risk associated with both high and low levels of physical activity. This finding aligns with prior research indicating that engaging in moderate physical activity diminishes the risk of falls and consequently reduces the incidence of hip fractures (18), whereas both sedentary behavior and excessive physical exertion may elevate it due to an increased likelihood of falls or stress fractures. Elevated FSH levels and decreased psoas muscle volume have been proposed as emerging risk factors for hip fractures in the older adults population (19, 20). However, research examining the association between MET and hip fracture remains limited. Our SHAP analysis demonstrates a non-linear relationship between MET and hip fracture risk, providing new insights into the intricate relationship between activity levels and fracture susceptibility. In our study, age emerged as a significant predictor, aligning with the existing literature that highlights the increased susceptibility of older adults to hip fractures, attributable to age-related declines in intrinsic foot muscle function and diminished postural stability (21). The robust correlation observed between fall incidents and hip fractures in our study reflects the well-documented relationship between falls and osteoporotic fractures, highlighting the critical importance of implementing fall prevention strategies among older adults (22). The inclusion of cognitive function scores, sleep duration, and marital status as predictors introduces novel perspectives. The association between cognitive decline and hip fractures may reflect the impact of cognitive impairment on gait and balance, increasing fall risk (23). The influence of sleep patterns suggests that sleep quality and duration can modulate fracture risk, potentially through effects on bone metabolism and daytime alertness (24). Marital status as a predictor could indicate the social support and lifestyle factors associated with living alone or with a partner, influencing health behaviors and fall risk (25). Our study diverges from previous research by incorporating a broad range of sociodemographic and lifestyle factors into a machine learning framework (26). Unlike tools like FRAX and Garvan FRC (27), which focus on clinical indicators and bone mineral density, our model leverages routinely collected CHARLS data, enhancing accessibility and affordability for risk assessment in non-Western populations.

Despite the robustness of our model, several limitations must be acknowledged. The reliance on self-reported data for hip fracture outcomes is susceptible to recall bias and misclassification bias, potentially affecting the accuracy of our results. Cognitive decline or other health issues may impair individuals’ ability to accurately recall or report their hip fracture history. Moreover, the cross-sectional nature of the CHARLS data limits our ability to establish causality or temporality in the observed associations. To address these limitations, future research should consider longitudinal studies to confirm the temporal relationships and causality between the identified risk factors and hip fracture incidence. Additionally, validating and adapting the model in different populations and settings could enhance its generalizability and applicability worldwide. In addition, model performance may be affected by the quality and breadth of data available in the CHARLS dataset. Missing data, while resolved by median interpolation, may still introduce bias or affect the predictive accuracy of the model. More importantly, its excellent performance on a completely independent external validation cohort (AUC = 0.89) provides robust evidence of its generalizability and argues against overfitting. Finally, it is important to contextualize the Brier score. The low score of 0.0103, while indicating excellent accuracy, is partly an expected result in a dataset with a very low event rate (2.9%); nonetheless, it represents a substantial improvement over a simple model predicting the base prevalence.

The results of this study hold significant implications for clinical practice, healthcare policy, and future research, particularly in the context of China’s aging population. For clinicians, the prediction model formulated in this research offers a valuable instrument for the early identification of individuals at elevated risk of hip fracture (28). By integrating modifiable lifestyle factors, including physical activity levels (measured in METs), cognitive function, and sleep patterns, clinicians are equipped to initiate discussions about targeted interventions. It is crucial to emphasize that this model is a risk stratification tool, not a diagnostic one. Any clinical application would require further large-scale prospective validation and impact assessment to confirm its utility in real-world settings. For instance, individuals exhibiting low MET values may gain from a tailored exercise regimen aimed at enhancing muscular strength and balance (29), thereby mitigating the risk of falls. Conversely, individuals with elevated MET values might require expert guidance to prevent excessive physical exertion, which could increase the likelihood of falls. Advanced age has been identified as a significant risk factor, due in large part to pelvic joint stiffness and reduced trunk motor control, which underscores the importance of adopting proactive fall prevention strategies in older adults (30–32). The capacity to predict the risk of hip fractures holds significant implications for health policy. By enabling the targeted allocation of resources to high-risk populations, it has the potential to alleviate the overall burden on the healthcare system (33, 34). This study establishes a basis for future research on hip fracture risk factors and the advancement of predictive models. The machine learning methods employed here offer opportunities to enhance risk prediction by incorporating genetic markers, imaging, and longitudinal health records (35–37). Future work could also involve validating the model across diverse populations to evaluate its generalizability.

## 5 Conclusion

In summary, the predictive model formulated in this study serves as a promising instrument for clinicians and public health officials in identifying individuals at elevated risk for hip fracture over a 7-year period. By integrating readily available and modifiable lifestyle factors, the model provides a scientific basis for developing early intervention strategies, which may ultimately lead to a reduction in hip fracture incidence. While our machine learning-based predictive model represents a significant advancement, we stress that it requires further prospective validation before it can be considered for clinical implementation. Our study highlights the multifaceted nature of hip fracture risk and the potential for lifestyle changes to reduce this risk. Our study highlights the multifaceted nature of hip fracture risk and the potential for lifestyle changes to reduce this risk, contributing to the growing body of research at the intersection of machine learning and public health.

## Data Availability

The CHARLS dataset used in this study is publicly available from the official CHARLS website (http://charls.pku.edu.cn/). Detailed definitions of the variables used in the final model and the model's hyperparameters are provided in the Supplementary materials (Supplementary Document S1). The Python code used for the analysis is available from the corresponding author upon reasonable request.

## Footnotes

1
http://charls.pku.edu.cn/
