# Supplementary material for: Machine learning approaches to predict hip fracture incidence: insights from the CHARLS dataset
Source: Front Public Health. 2026 Jan 13;13:1624843. doi: 10.3389/fpubh.2025.1624843 (PMC12834802; doi:10.3389/fpubh.2025.1624843)
Supplement: Supplementary file 1 [file Data_Sheet_1.docx]

**Supplementary Document S1: Variable Definitions and Model Hyperparameters**

| **Variable** | **Definition** | **Type** | **Coding / Units** |
| --- | --- | --- | --- |
| ****MET**** (Metabolic Equivalent of Task) | A continuous variable representing the total weekly physical activity level, calculated using the standard CHARLS formula. | Continuous | MET-minutes/week |
| ****Age**** | Participant's age at the time of the 2011 baseline survey. | Continuous | Years |
| ****Fall Down**** | A binary variable indicating whether the participant had experienced a fall in the year preceding the baseline interview. | Categorical | 1: Yes; 0: No |
| ****Drinking**** | A categorical variable describing the participant's alcohol consumption status. | Categorical | 0: Never; 1: Current; 2: Quit |
| ****Cognitive Function Scores**** | A composite score assessing episodic memory (immediate and delayed word recall) and mental status (based on the Telephone Interview for Cognitive Status, TICS-10). | Continuous | Points (Higher scores indicate better cognitive function) |
| ****Sleep duration of nap after lunch**** | The self-reported duration of the participant's typical afternoon nap. | Continuous | Minutes |
| ****Residence**** | The participant's area of residence. | Categorical | 1: Urban; 0: Rural |
| ****Total sleep duration**** | The self-reported total sleep duration over a 24-hour period (night sleep + nap). | Continuous | Minutes |
| ****Marital Status**** | The participant's current marital status at baseline. | Categorical | 1: Married; 2: Separated/Divorced/Widowed; 3: Unmarried |

**Final Hyperparameters for the Optimized Random Forest Model**

| **Hyperparameter** | **Value** | **Description** |
| --- | --- | --- |
| **n_estimators** | 200 | The number of decision trees in the forest. |
| **max_depth** | 15 | The maximum depth of each decision tree. This helps control model complexity. |
| **min_samples_split** | 2 | The minimum number of samples required to split an internal node. |
| **min_samples_leaf** | 5 | The minimum number of samples required to be at a leaf node. This helps smooth the model. |
| **class_weight** | 'balanced' | Automatically adjusts weights inversely proportional to class frequencies. This was crucial for addressing the class imbalance issue. |
| **random_state** | 88 | A fixed seed value to ensure the reproducibility of results. |
| **criterion** | 'gini' | The function to measure the quality of a split (Gini impurity). |
