# Supplementary figures and images for: Machine learning approaches to predict hip fracture incidence: insights from the CHARLS dataset
Source: Front Public Health. 2026 Jan 13;13:1624843. doi: 10.3389/fpubh.2025.1624843 (PMC12834802; doi:10.3389/fpubh.2025.1624843)

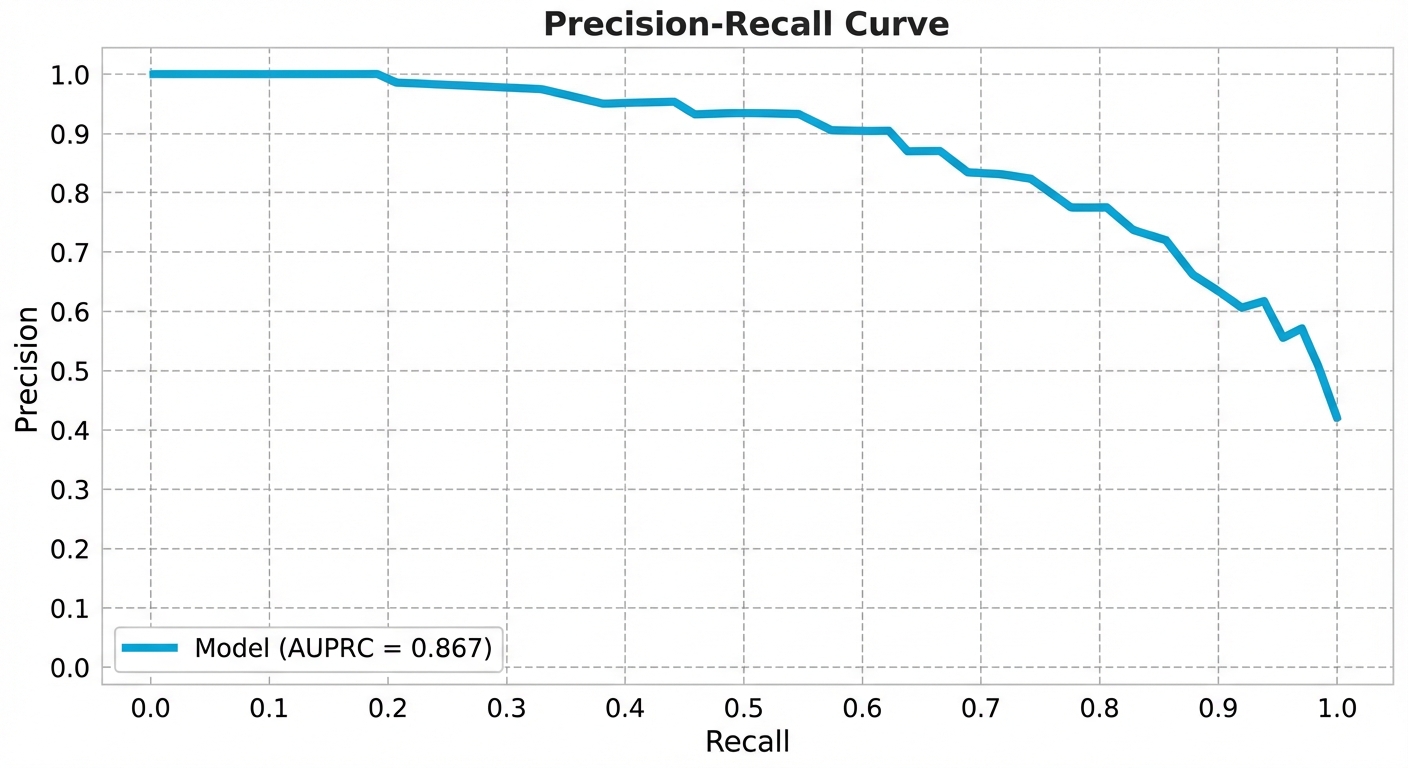

Supplement: Supplementary file 2 [file Image_1.PNG]

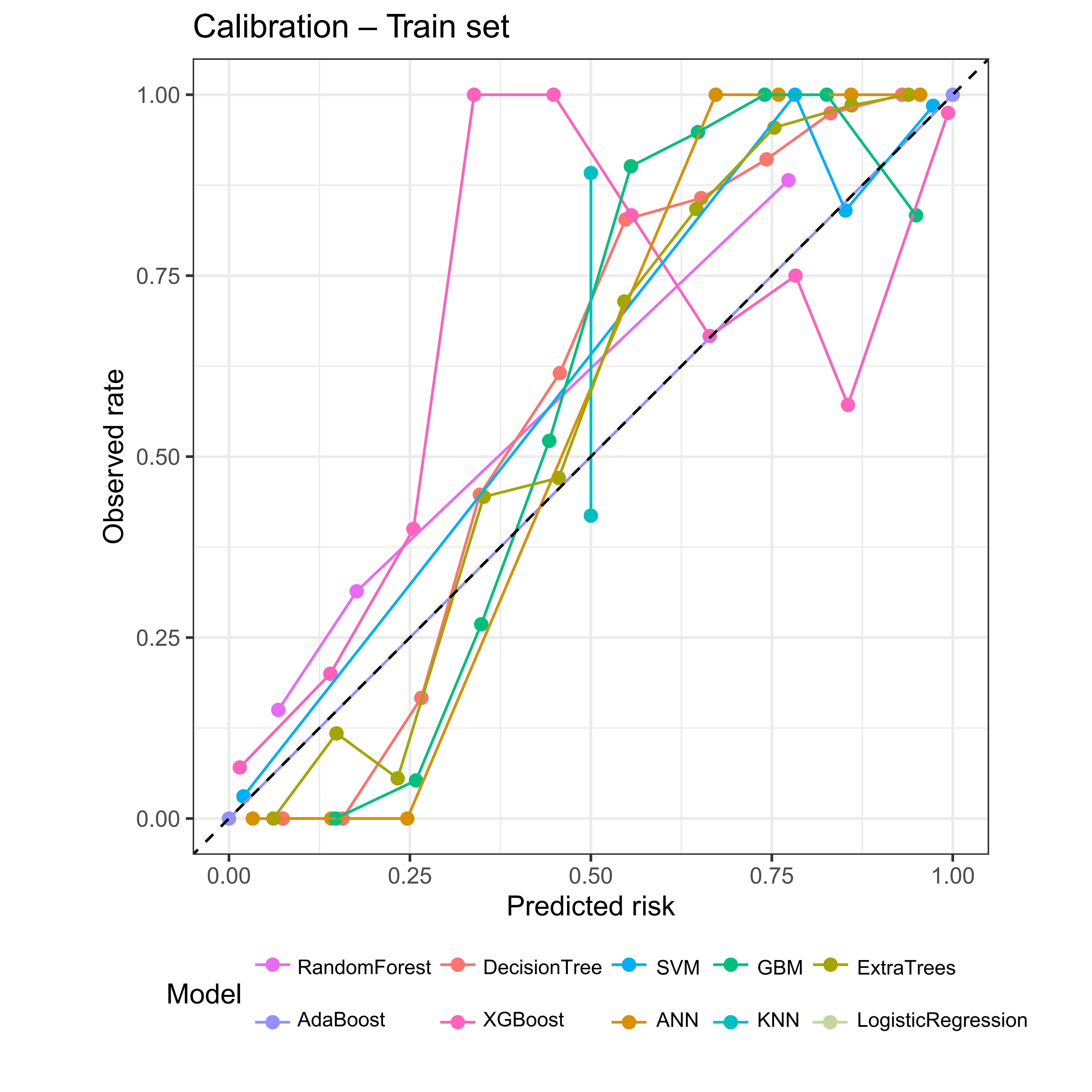

Supplement: Supplementary file 3 [file Image_2.PNG]
